# The positivity rates and drug resistance patterns of *Mycobacterium tuberculosis* using nucleotide MALDI-TOF MS assay among suspected tuberculosis patients in Shandong, China: a multi-center prospective study

**DOI:** 10.3389/fpubh.2024.1322426

**Published:** 2024-01-18

**Authors:** Xusheng Gao, Tongxia Li, Wenge Han, Yu Xiong, Shiyang Xu, Hongbao Ma, Qing Wang, Qiuxia Zhang, Guofeng Yang, Dan Xie, Peipei Jiang, Hailiang Wu, Mei Lin, Min Liu, Mingde Ni, Decui Wang, Ying Li, Lunxian Jiao, Caihong Ding, Zhongfa Zhang

**Affiliations:** ^1^Department of Tuberculosis, Shandong Public Health Clinical Center, Shandong University, Jinan, Shandong, China; ^2^Department of Tuberculosis, Qingdao Chest Hospital, Qingdao, Shandong, China; ^3^Department of Tuberculosis, Weifang Second People's Hospital, Weifang, Shandong, China; ^4^Department of Tuberculosis, Dezhou Second People's Hospital, Dezhou, Shandong, China; ^5^Department of Tuberculosis, Yantai Pulmonary Hospital, Yantai, Shandong, China; ^6^Department of Internal Medicine, Zaozhuang Tumor Hospital, Zaozhuang, Shandong, China; ^7^Department of Tuberculosis, Liaocheng Infectious Disease Hospital, Liaocheng, Shandong, China; ^8^Department of Respiratory Medicine, Tai'an Tumor Prevention and Treatment Hospital, Tai'an, Shandong, China; ^9^Department of Tuberculosis, Linyi People's Hospital, Linyi, Shandong, China; ^10^Department of Tuberculosis, Binzhou Central Hospital, Binzhou, Shandong, China; ^11^Department of Internal Medicine, Zibo First Hospital, Zibo, Shandong, China; ^12^Third Department of Respiratory Medicine, Yantai Beihai Hospital, Yantai, Shandong, China; ^13^Respiratory Center, Shandong Public Health Clinical Center, Shandong University, Jinan, Shandong, China

**Keywords:** tuberculosis, Shandong Province, nucleotide MALDI-TOF MS, drug resistance, mutation

## Abstract

**Objective:**

To investigate the positivity rates and drug resistance characteristics of *Mycobacterium tuberculosis* (MTB) among suspected tuberculosis (TB) patients in Shandong Province, the second-largest population province in China.

**Methods:**

A prospective, multi-center study was conducted from April 2022 to June 2023. Pathogen and drug resistance were identified using nucleotide matrix-assisted laser desorption ionization time-of-flight mass spectrometry (nucleotide MALDI-TOF MS).

**Results:**

Of 940 suspected TB patients included in this study, 552 cases were found to be infected with MTB giving an overall positivity rate of 58.72%. Total of 346 cases were resistant to arbitrary anti-TB drug (62.68%), with Zibo (76.47%), Liaocheng and Weihai (both 69.23%) ranking top three and TB treatment history might be a related factor. Monoresistance was the most common pattern (33.53%), with isoniazid the highest at 12.43%, followed by rifampicin at 9.54%. Further analysis of gene mutations conferring resistance revealed diverse types with high heteroresistance rate found in multiple anti-TB drugs.

**Conclusion:**

A relatively high rate of MTB positivity and drug resistance was found in Shandong Province during and after the COVID-19 pandemic, indicating the need for strengthening rapid identification of species and drug resistance among suspected TB patients to guide better medication and minimize the occurrence of drug resistance.

## 1 Introduction

Tuberculosis (TB), caused by *Mycobacterium tuberculosis* (MTB), remains a global public health event affecting public health worldwide. Approximately 10 million people are still afflicted with TB each year, resulting in around 1.5 million deaths. China continues to be a high-burden country for TB. According to the 2022 World Health Organization (WHO) Tuberculosis Report, over one-third of the TB cases in 2021 were concentrated in India and China, accounting for 28 and 7.4%, respectively (1). The situation for TB prevention and control has become more severe with the emergence and spread of drug-resistant strains. Treatment of drug-resistant TB (DR-TB) is prolonged, comes with significant side effects, and poses a heavy economic burden (2). Moreover, with extension of treatment time, the risk of infection with non-tuberculous mycobacteria (NTM) also increases (3). Rapid and accurate diagnosis of MTB infection and drug resistance will contribute to the timely implementation of precise treatment and control of TB transmission.

Shandong Province is located in the eastern coastal region of China, with a population of over 100 million, ranked second-most populous province. In 1992, Shandong Province began to implement the WHO-recommended directly observed treatment, short-course (DOTS) strategy for TB control. By 1997, the DOTS strategy had achieved full coverage at the county level in Shandong Province. The incidence rate of TB decreased from 40.8 per 100,000 in 2005 to 26.25 per 100,000 in 2017 (4). The reported incidence of MTB per 100,000 people in Shandong was 20.60 in 2022. According to a study by Song et al. in 2018, the prevalence rate of mono-resistant TB (MR-TB), multidrug-resistant TB (MDR-TB), and extensively drug-resistant TB (XDR-TB) among newly diagnosed TB patients in Shandong Province were 13.35, 3.73, and 4.30%, respectively (5).

Although several published studies have described the epidemiology of TB in Shandong Province (4, 5), there is still limited prospective research on the prevalence and characteristics of TB and DR-TB in recent years, particularly during and after the COVID-19 pandemic. As the second-largest populous province in China, it is necessary to comprehensively characterize the recent trends and features of TB and DR-TB in Shandong Province, providing a theoretical basis for better prevention and control of TB and DR-TB in this region.

The matrix-assisted laser desorption ionization time-of-flight mass spectrometry based on nucleic acid extracted from clinical samples (nucleotide MALDI-TOF MS) is a novel molecular diagnostic technology (6). By performing multiple PCR-specific amplifications of target gene segments and undergoing single nucleotide extension reactions, the reaction product is combined with matrix, and a mass spectrum is obtained finally, providing information on the species and drug resistance. Several studies have confirmed the high accuracy of nucleotide MALDI-TOF MS in identifying MTB and its drug resistance (6, 7). In recent years, this technique has been highly favored by clinicians due to its high accuracy, high throughput, rapidity, high resolution and low cost. Recently, China has also issued an expert consensus on the application of nucleotide MALDI-TOF MS in TB and NTM infection diseases (8), further recognizing the diagnostic capability of this technology.

In this study, we prospectively enrolled suspected TB patients from 12 designated TB hospitals in Shandong Province. Nucleotide MALDI-TOF MS on clinical samples from participants was applied for identification of MTB and its resistance to anti-TB drugs and basic information of them were simultaneously collected. Based on the results of nucleotide MALDI-TOF MS, we analyzed and characterized the positivity rates of TB and its drug resistance patterns among suspected TB patients in Shandong Province, providing theoretical support for the prevention and control of TB in Shandong Province.

## 2 Methods

### 2.1 Study design and study population

This is a prospective, cross-sectional, multi-center study conducted in Shandong Province, which is located in the eastern coastal region of China, bordered by the Bohai Sea and the Yellow Sea and composed of 16 prefecture-level cities, with an area of 157,100 square kilometers. Shandong Province has the second-largest population in China with over 100 million residents. Twelve designated TB hospitals participated in this study, including Public Health Clinical Center Affiliated to Shandong University, Yantai Pulmonary Hospital, Zibo First Hospital, Dezhou Second People's Hospital, Liaocheng Infectious Disease Hospital, Linyi People's Hospital, Qingdao Chest Hospital, Yantai Beihai Hospital, Zaozhuang Tumor Hospital, Binzhou Central Hospital, Tai'an Tumor Prevention and treatment Hospital, and Weifang Second People's Hospital. Supplementary Figure 1 displays the cities where the 12 participated hospitals are located. Considering Publich Health Clinical Center Affiliated to Shandong University is the largest TB designated hospital in Shandong Province, it nearly carries more than half of the TB patients' diagnosis and treatment, with many patients from other cities referring to the hospital themselves or being referred to the hospital. The other 11 hospitals are also responsible for most of the TB patients in their own city and the neighboring cities. Therefore, the population enrolled in this study could well represent the whole Shandong Province. Prior to the study, we provided standardized training to the principles of the 12 participating hospitals, including the purpose of this study, the procedures, sample handling, etc., to ensure, as far as possible, uniformity of operation across hospitals. All patients included in the study were seen at TB department specialized for TB care.

During the study period, trained and experienced clinicians conducted screening based on symptoms and signs of suspected TB and obtained informed consent from eligible individuals who met the inclusion criteria. Each participant was required to provide at least one sample, which was sent to Shanghai Conlight Medical Co., Ltd. for nucleotide MALDI-TOF MS assay. Basic information of each participant, including demographic and clinical characteristics, was collected simultaneously. Inclusion criteria: (1) individuals with suspected symptoms and signs of TB; (2) no severe illnesses; (3) no antibiotic use within the past 6 months; (4) willingness to participate in the study and sign the informed consent. Exclusion criteria: (1) presence of severe illnesses; (2) inability to provide informed consent. The severe illnesses in our study were defined by the presence of severe shortness of breath, significant impairment of daily activities and the high risk of death associated with a particular disease or condition.

### 2.2 Ethical approval

The study has obtained approval from the Ethics Committee of Public Health Clinical Center Affiliated to Shandong University (No. GWLCZXEC2022-74). All participants were provided informed consent, and all data were kept confidential. Research involving human subjects compiled with all relevant national regulations and is in accordance with the tenets of the Helsinki Declaration.

### 2.3 Sample size

Sample size was estimated according to the cross-sectional study type by taking MTB positivity rate among suspected TB patients in Shandong Province at 50%, with DR-TB accounting for about 50% of TB patients, 5% margin of error (*d* = 0.05) and 95% confidence interval (*z* = 1.96). The sample size was 922 by considering a 20% non-response rate. The final sample size we included was 940 based on our actual situation.

### 2.4 Sample collection

Enrolled patients were asked to provide eligible samples at any time within 7 days after their visit at hospital. The sample types included but were not limited to sputum, bronchoalveolar lavage fluid (BALF), pus, tissue, cerebrospinal fluid (CSF), and puncture fluid. Experienced clinicians collected these samples according to the requirements and performed appropriate processing procedures based on the sample type, preserving them in sample preservation solution. All of the samples were then sent to Shanghai Conlight Medical Co., Ltd. according to the sample transportation requirements for subsequent processing and testing.

### 2.5 Nucleotide MALDI-TOF MS detection

Nucleotide MALDI-TOF MS detection to assess the mycobacterial species and MTB drug sensitivity was based on Conlight TB&DR assay (7). After receiving the clinical specimens, the following experimental steps were conducted: (1) Sample DNA extraction, strictly following the manufacturer's instructions of the kit used; (2) PCR amplification, preparing PCR mix and adding it to the 96-well plate, followed by adding to the DNA template and running the PCR program accordingly; (3) Alkaline phosphatase (SAP) reaction, preparing the SAP mix and adding it to the 96-well plate to remove residual deoxynucleotide triphosphates; (4) Extension reaction, preparing extension primers and iPLEX extension mix, adding to the 96-well plate, and performing the iPLEX extension reaction; (5) Sample desalting and loading it into the chip plate for mass spectrometry detection in the instrument; (6) Analyzing the results of mass spectrometry. For each specific steps, the concrete information can be referred to the citation (7). The information on drug resistance associated gene loci designed by Shanghai Conlight Medical Co., Ltd. is provided in Supplementary Table 1.

### 2.6 Data collection and statistical analysis

Date analysis was performed using R software (R 4.2.0 version). Demographic and clinical information of the included patients were recorded in EXCEL spreadsheets and assessed with descriptive statistics. Continuous data were expressed as average with standard errors, whereas categorical data were expressed as numbers and percentages. For intergroup comparisons of categorical variables, chi-square test was carried out; for continuous variables, the Mann-Whitney *U*-test was employed. A two-tailed *p*-value of < 0.05 was considered statistically significant.

## 3 Results

### 3.1 Characteristics of enrolled participants

**Figure 1:**
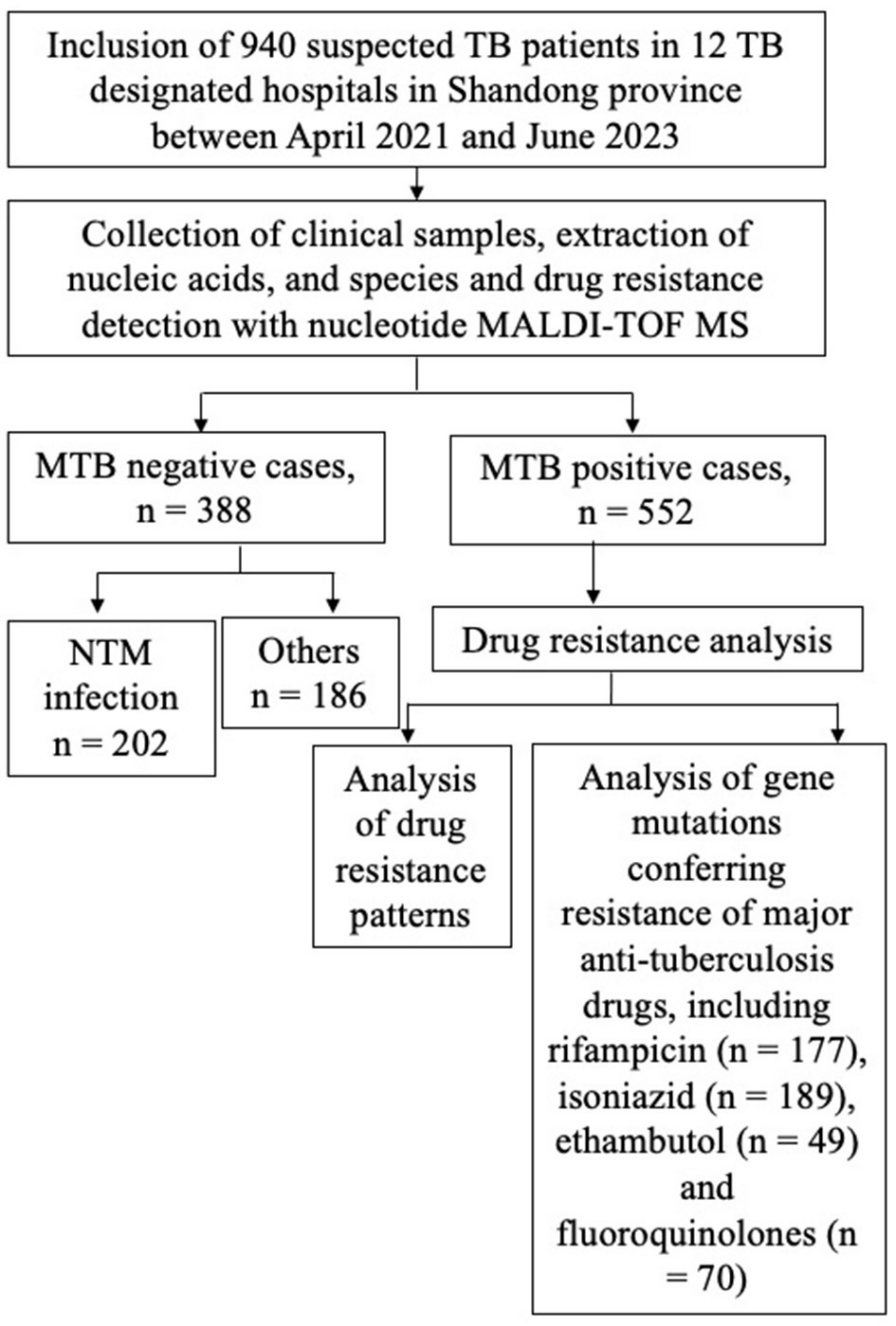
Flow diagram of the study design.

The flowchart of the study design is shown in Figure 1. In order to study the MTB positivity rates and characteristics of TB and DR-TB in Shandong Province, we prospectively included 940 suspected TB patients from 12 designated TB hospitals between April 2021 and June 2023, based on the inclusion criteria. Table 1 presents the basic information of the study population. Nearly two-thirds were male (63.09%), with an average age of 48.59 years (95% CI: 47.35–49.83 years), and an overall distribution was uniform across all ages, with the largest number of people in the 46–65 years (33.09%). A previous TB treatment history was found in 35.74% (336/940) of enrolled patients. The samples collected were diverse, with sputum being the most common (44.89%), followed by BALF samples at 40.85%, and tissue samples with 60 cases (6.38%). Other samples included pleural fluid (2.13%), pus (1.60%), nine cases of CSF, four cases of puncture fluid, and only three and two cases of urine and ascites, respectively. Based on the identification results by nucleotide MALDI-TOF MS assay, 552 cases (58.72%) were infected with MTB, classified as TB patients, with the remaining as non-TB patients (41.28%, 388/940).

**Table 1:** Basic information of study population.

**Categories**	**Cases (*n*)**	**Percent (%)**
**Gender**		
Male	593	63.09
Female	347	36.91
**Age**		
~25	149	15.85
26–45	261	27.77
46–65	311	33.09
66~	219	23.30
**TB treatment history**		
Yes	336	35.74
No	604	64.26
**MTB results of nucleotide MALDI-TOF MS**		
Positive (+)	552	58.72
Negative (-)	388	41.28
**Sample types**		
BALF	384	40.85
Sputum	422	44.89
Pus	15	1.60
Tissue	60	6.38
Pleural fluid	20	2.13
Ascitic fluid	2	0.21
Urine	3	0.32
CSF	9	0.96
Puncture fluid	4	0.43
Culture	21	2.23

MTB, Mycobacterium tuberculosis; MALDI-TOF MS, matrix-assisted laser desorption ionization time-of-flight mass spectrometry; BALF, bronchoalveolar lavage fluid; CSF, Cerebrospinal fluid.

### 3.2 Characteristics of TB patients in Shandong Province

In order to characterize the basic features of TB patients in Shandong Province, we further compared the demographic and relevant clinical characteristics between TB patient and non-tuberculosis (non-TB) patients in the study population. There were significant differences between the TB group and non-TB group in terms of age, gender, comorbid diabetes, and radiological findings (Table 2). The TB group had a higher proportion of male patients and individuals in the younger to middle-aged group. Among TB group, 14.67% of patients had diabetes, while only 7.73% of cases were found in non-TB group. In terms of chest radiograph features, 34.96% of patients in TB group had cavities, where 25.52% in non-TB group. Additionally, 14.86% of patients were simultaneously infected with NTM in TB group, while 52.06% of cases had NTM infection in non-TB group, suggesting NTM infection is an important confounding factor in suspected TB patients where MTB is not detected.

**Table 2:** Demographic and clinical characteristics between the TB and non-TB groups.

**Characteristics**	**TB group *n* = 552**	**Non-TB group *n* = 388**	***p-*value**
**Gender (** ***n*** **, %)**			0.004
Male	369 (66.85%)	224 (57.73%)	
Female	183 (33.15%)	164 (42.27%)	
**Age, years (mean, 95% CI)**	45.62 (43.98–47.25)	52.81 (50.99–54.63)	< 0.001
**Diabetes (** ***n*** **, %)**			0.001
Yes	81 (14.67)	30 (7.73)	
No	471 (85.32)	358 (92.23)	
**Farmer (** ***n*** **, %)**			0.108
Yes	222 (40.22)	136 (35.05)	
No	330 (59.78)	252 (64.95)	
**NTM infection (** ***n*** **, %)**			< 0.001
Yes	82 (14.86%)	202 (52.06%)	
No	470 (85.14%)	186 (47.94%)	
**Cavity (** ***n*** **, %)**			0.002
Yes	193 (34.96)	99 (25.52)	
No	359 (65.04)	289 (74.48)	

TB, tuberculosis; NTM, non-tuberculous mycobacterium; CI, confidence intervals.

### 3.3 Description of DR-TB characteristics and patterns in Shandong Province

DR-TB has always been a challenge in the prevention and treatment of TB. Therefore, we further conducted a detailed description of the drug resistance status in the identified group of TB patients in this study. According to the results of nucleotide MALDI-TOF MS assay, MTB strains from 206 cases (37.32%) didn't show any mutations in the detected drug resistance associated gene loci, indicating drug susceptible TB (DS-TB) and the remaining 346 (62.68%) TB patients were resistant to at least one anti-TB drug, classified as DR-TB. Here, we compared the clinical characteristics of DR-TB and DS-TB patients (Table 3). Both groups showed no significant differences in terms of age, gender, cavities, diabetes, or occupation as farmers (Table 3). Only the previous TB treatment history was associated with drug resistance (*p* = 0.040; Table 3).

**Table 3:** Demographic and clinical characteristics between DR-TB and DS-TB groups.

**Characteristics**	**DR-TB *n* = 346**	**DS-TB *n* = 206**	***p*-value**
**Age (** ***n*** **, %)**			0.282
≤ 30	83 (23.99%)	62 (30.10%)	
30–50	103 (29.77%)	58 (28.16%)	
≥50	160 (46.24%)	86 (41.75)	
**Gender (** ***n*** **, %)**			0.750
Male	233 (67.34%)	136 (66.02%)	
Female	113 (32.66%)	70 (33.98%)	
**TB treatment history (** ***n*** **, %)**			0.040
Yes	134 (38.73%)	62 (31.63%)	
No	212 (61.27%)	144 (69.90%)	
**Cavity (** ***n*** **, %)**			0.139
Yes	129 (37.28)	64 (31.07)	
No	217 (62.72)	142 (68.93)	
**Farmer (** ***n*** **, %)**			0.112
Yes	148 (42.77)	74 (35.92)	
No	198 (57.23)	132 (64.08)	
**Diabetes (** ***n*** **, %)**			0.121
Yes	57 (16.47)	24 (11.65)	
No	289 (83.53)	182 (88.35)	

DR-TB, drug-resistant tuberculosis; DS-TB, drug susceptible TB.

DR-TB in Shandong Province displayed diverse patterns of drug resistance, ranging from resistance to a single drug to over five drugs (Table 4). Mono-resistant TB was the most common, with a total of 116 cases, accounting for 33.53% (116/346) of all DR-TB cases. Among them, isoniazid mono-resistant TB was the most prevalent, accounting for 12.43% (43/346), followed by rifampicin mono-resistant TB (9.54%, 33/346), and fluoroquinolones mono-resistant TB at 5.20% (18/346). There were 74 cases (21.39%) of dual-drug resistance, the same as triple-drug resistance. In the dual-drug resistant cases, resistance to rifampicin combined with isoniazid was the most common, with 26 cases, followed by resistance to rifampicin combined with streptomycin, with 11 cases. In the triple-drug resistant cases, resistance to injectable drugs (amikacin + kanamycin + capreomycin) was the most common, with 36 cases. There were 37 cases (10.69%) of four-drug resistant TB and 45 cases (13.01%) of resistance to five or more drugs.

**Table 4:** Drug-resistance types in DR-TB.

**Types**	**Cases (*n*)**	**Percent (%)**
**Single-drug resistance**	**116**	**33.53**
R	33	9.54
H	43	12.43
E	5	1.45
Z	3	0.87
FQs	18	5.20
S	8	2.31
PAS	4	1.16
Cs	2	0.58
**Double-drug resistance**	**74**	**21.39**
R+H	26	7.51
R+S	11	3.18
R+FQs	7	2.02
R+Km	1	0.29
H+S	10	2.89
H+E	3	0.87
H+FQs	5	1.45
H+Cs	1	0.29
H+PAS	1	0.29
E+S	1	0.29
FQs+S	2	0.58
FQs+Lzd	1	0.29
FQs+Cs	2	0.58
Z+PAS	1	0.29
BDQ+CFZ	2	0.58
**Triple-drug resistance**	**74**	**21.39**
R+H+S	18	5.20
R+H+E	9	2.60
R+H+FQs	2	0.58
R+H+PAS	2	0.58
R+E+S	1	0.29
R+E+FQs	1	0.29
H+E+FQs	3	0.87
H+FQs+PAS	2	0.58
Am+Km+Cm	36	10.40
**Tetra-drug resistance**	**37**	**10.69**
R+H+E+S	10	2.89
R+H+E+FQs	2	0.58
R+H+S+FQs	9	2.60
R+H+S+Cs	1	0.29
R+Am+Km+Cm	6	1.73
H+Am+Km+Cm	5	1.45
S+Am+Km+Cm	1	0.29
E+Am+Km+Cm	1	0.29
FQs+Am+Km+Cm	1	0.29
Cs+Am+Km+Cm	1	0.29
**Five more drug-resistance**	**45**	**13.01**

R, rifampicin; H, isoniazid; Z, pyrazinamide; E, ethambutol; FQs, fluoroquinolones; S, streptomycin; PAS, p-aminosalicylate; Am, amikacin; Km, kanamycin; Cm, capreomycin; Cs, cycloserine; CFZ, clofazimine; BDQ, bedaquiline; LZD, linezolid.

Analyzing the resistance to any single anti-TB drug among DR-TB in Shandong Province (Table 5), we found that isoniazid resistance was the highest (54.62%, 189/346), followed by rifampicin (51.16%, 177/346), and resistance to infectable drugs, including streptomycin, kanamycin, capreomycin and amikacin, was also relatively high, all above 25%. Of note, the resistance rate to fluoroquinolones, a core group of drugs for DR-TB, reached 20.23% (40/346). Five cases of resistance to the new drug, bedaquiline, were also observed.

**Table 5:** Resistance to any of anti-TB drugs among DR-TB patients.

**Anti-TB drugs**	**Cases (*n*)**	**Percent (%)**
R	177	51.16
H	189	54.62
E	49	14.16
Z	5	1.45
S	96	27.75
FQs	70	20.23
Km	90	26.01
Cm	89	25.72
Am	88	25.43
PAS	12	3.47
Cs	13	3.76
BDQ	5	1.45
CFZ	5	1.45
LZD	2	0.58

R, rifampicin; H, isoniazid; Z, pyrazinamide; E, ethambutol; FQs, fluoroquinolones; S, streptomycin; PAS, p-aminosalicylate; Am, amikacin; Km, kanamycin; Cm, capreomycin; Cs, cycloserine; CFZ, clofazimine; BDQ, bedaquiline; LZD, linezolid.

### 3.4 Geographical distribution of MTB arbitrary drug resistance rates in Shandong Province

**Figure 2:**
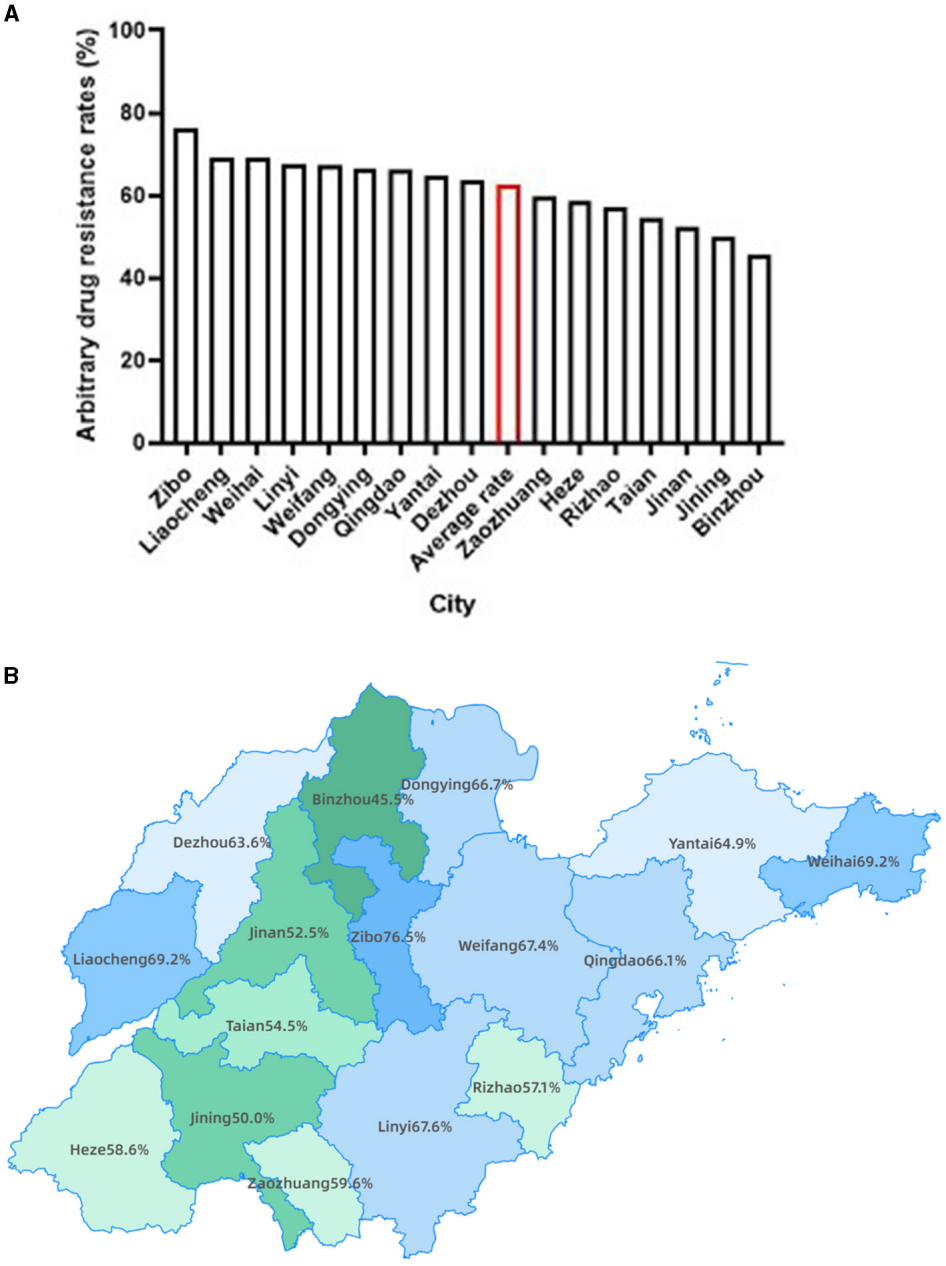
The distribution of MTB arbitrary drug resistance rates in Shandong province. **(A)** Bars presenting the MTB arbitrary drug resistance rate for each city of Shandong province, as well as the average rate of Shandong province highlighted with red. **(B)** Geographic distribution of MTB arbitrary drug resistance rate by city.

We further analyzed the MTB arbitrary drug resistance rates in the 16 prefecture-level cities of Shandong Province. The results showed that more than half of the cities had MTB arbitrary drug resistance rates higher than the average rate of Shandong Province (Figures 2A, B), with Zibo the highest (76.47%), followed by Liaocheng and Weihai (both 69.23%). Interestingly, these three cities are geographically located in the center, west and east respectively, with no concentration in one region (Figure 2B). Conversely, cities with lower MTB arbitrary drug resistance rates than the provincial average were concentrated in the southwestern of Shandong Province, with Binzhou lowest (45.45%), followed by Jining (50.00%) and Jinan (52.50%) (Figures 2A, B).

### 3.5 Molecular characteristics of drug-resistant MTB in Shandong Province

Finally, we compiled the molecular characteristics of all DR-TB isolates (refer to Supplementary Table 2 for details). Below, we provide a detailed description of the molecular characteristics of resistance to rifampicin, isoniazid, ethambutol, and fluoroquinolones.

**Figure 3:**
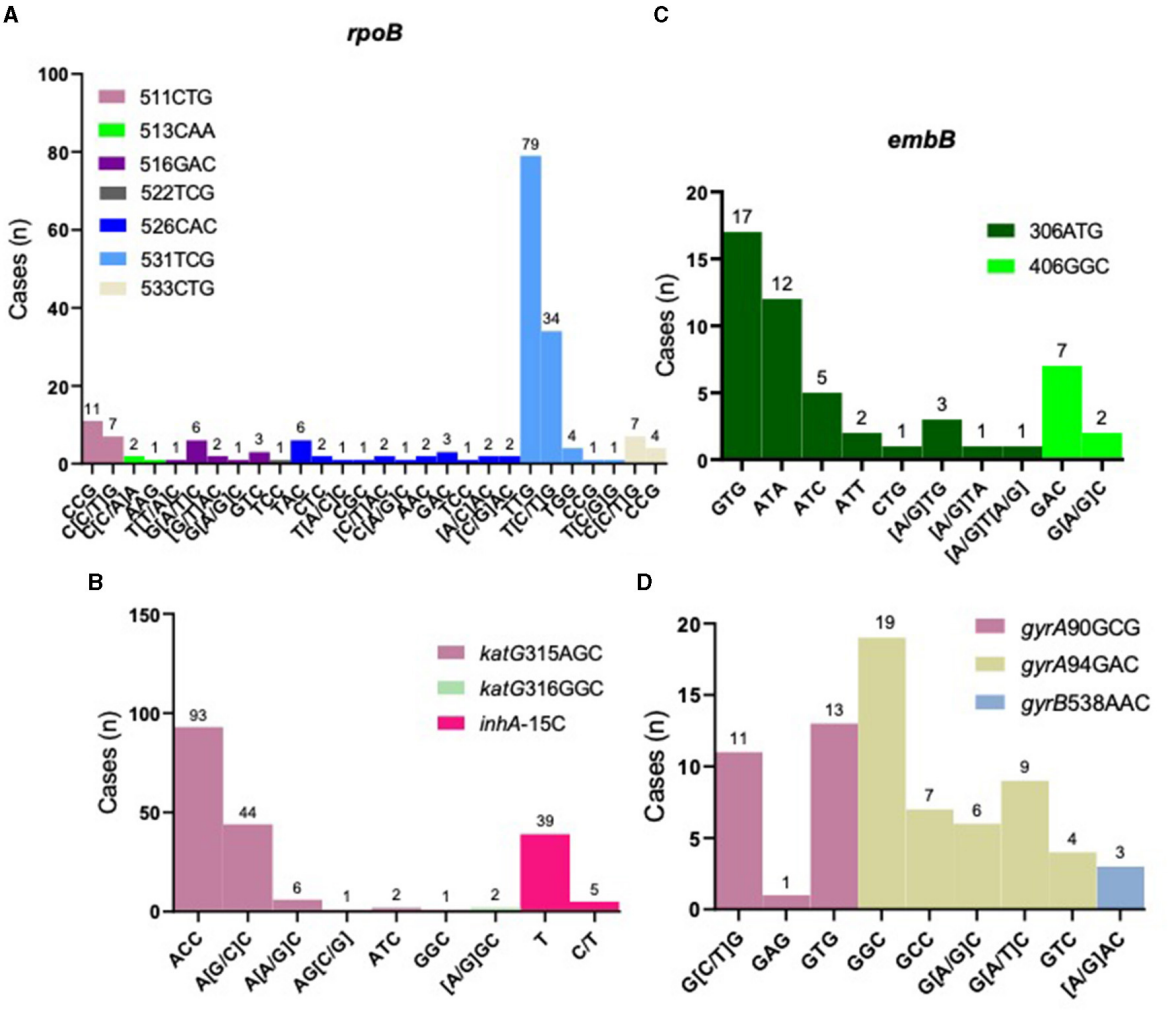
Distribution of resistance-related gene loci. Distribution of resistance loci mutations for rifampicin resistance-related gene, *rpoB* **(A)**; isoniazid resistance-related genes, *katG* and *inhA* **(B)**; ethambutol resistance-related gene, *embB* **(C)** and fluoroquinolones resistance-related genes, *gyrA* and *gyrB* **(D)**.

Rifampicin: Rifampicin resistance is mainly concentrated in the rifampicin resistance-determining region (RRDR) of *rpoB* gene. Among the 177 rifampicin-resistant strains, *rpoB* codon 531 had the highest percentage of mutations (67.23%, 119/177), with five types of mutations detected and TCG > TTG (Ser > Leu) being the most common. *rpoB* codon 526 was found in 24 rifampicin- resistant strains, with a total of 11 different mutation types (Figure 3A). Heteroresistance (co-existence of wild-type and mutant strains) was detected at codons 511, 516, 526, 531, and 533, among which, the most common was codon 531 TCG, with 34 cases. The proportion of rifampicin heteroresistance was 37.85% (67/177) (Supplementary Table 2). We further conducted a more extensive examination of the geographical distribution of rifampicin-resistant cases. Notably, Yantai exhibited the highest incidence with a total of 28 cases, followed closely by Weifang and Qingdao, with 22 and 20 cases, respectively. It is worth mentioning that these three cities are situated in close proximity to one another within the eastern region of Shandong Province. However, Liaocheng city, located in the western part of the province, also reported 20 cases displaying resistance to rifampicin (Supplementary Figure 2A).

Isoniazid: Isoniazid resistance is mainly concentrated in *katG* gene. Our results showed that *katG* codon 315 was the most common mutation conferring resistance, accounting for 77.78% (147/189), of which six types of mutations were found with AGC > ACC (Ser > Thr) being the most frequent. *inhA* C-15 mutation was found in 44 cases, representing 23.28% (44/189), two of which also harbored *katG* codon 315 mutation (Figure 3B and Supplementary Table 2). Isoniazid heteroresistance was present in 52 strains (27.51%, 52/189), with codon 315 AGC > AGC/ACC being the most prevalent (Supplementary Table 2).

Ethambutol: Ethambutol resistance is mainly associated with mutations in *embB* gene. Our results showed that *embB* codon 306 was the most frequent mutation, accounting for 85.71% (42/49), of which eight types of mutations were found, with ATG > GTG (Met > Val) being the most common (Figure 3C and Supplementary Table 2). Heteroresistance was detected in six cases (12.24%, 6/49) (Supplementary Table 2).

Fluoroquinolones: Resistance to fluoroquinolones is primarily associated with *gyrA* gene. Our results showed that *gyrA* codon 94 was the most frequent, accounting for 64.29% (45/70), of which five types of mutations were observed, with GCG > GGC (Ser > Leu) the most common (Figure 3D and Supplementary Table 2). Two strains with *gyrB* codon 538 mutation also harbored mutants in *gyrA* gene. Twenty-seven cases were found to have heteroresistance, possessing 38.57% (27/70) (Supplementary Table 2).

## 4 Discussion

Although there have been several studies reporting the prevalence and drug resistance of TB from various aspects in Shandong Province (5, 9, 10), most of them are retrospective studies, and the data were collected before the outbreak of the COVID-19 pandemic. Currently, there is still a lack of multi-center prospective studies characterizing the detection and drug resistance patterns of MTB in suspected TB patients in Shandong Province during and after the COVID-19 pandemic. In this study, we conducted a multi-center prospective study in Shandong Province to assess the infection rate and drug resistance of MTB in suspected TB patients based on nucleotide MALDI-TOF MS assay. Our results revealed an overall MTB positivity rate was 58.72% among suspected TB patients and relatively high proportion of DR-TB cases (62.68%) in Shandong Province during and after the COVID-19 pandemic, with Zibo, Liaocheng, and Weihai ranking top three.

The results of our study showed much higher MTB arbitrary drug resistance rate as compared with the previous report (5). There were several contributing factors. The period in which this study was conducted was during and at the end of the COVID-19 pandemic, where many patients were more difficult with access to a doctor, and most of them came to the hospital when their symptoms had progressed to the point where they were very obvious and difficult to control, with more complex. Secondly, many of the patients in our study had TB treatment history. To some extent, this study suggests that the COVID-19 pandemic might have some impact on TB drug resistance. Our findings of high MTB arbitrary drug resistance rates of TB patients in Shandong Province might partially attributed to the previous TB treatment history. It was further found by geographical distribution that more patients with TB treatment history existed in areas with high arbitrary drug resistance rates. We found cities with lower MTB arbitrary drug resistance rates than the provincial average were concentrated in the southwestern of Shandong Province, which might be related to the high MTB positivity rates in these regions. Here, we also analyzed the MTB positivity rates in the 16 prefecture-level cities of Shandong Province and found the cities with high levels of MTB positivity rates were concentrated in the southwestern of Shandong Province (Supplementary Figures 3A, B). Furthermore, we also analyzed the geographic distribution of TB patients resistant to three or more anti-TB drugs. The findings revealed that Yantai and Qingdao, located in the eastern part of Shandong Province, exhibited the highest incidence, followed by Dezhou in the west and Linyi, also in the east (Supplementary Figure 4). Based on the integrated data analysis of this study, a high TB positivity and drug resistance rate during and after the COVID-19 pandemic in Shandong Province can be noticed, which also give us some insights: (1) The timely diagnosis of TB is of utmost important, necessitating increased emphasis on the prompt detection of suspected TB cases to facilitate early diagnosis and treatment, thereby impeding its further transmission; (2) The assessment of TB patients' resistance to anti-TB drugs assumes significant important, particularly for key first-line drugs such as rifampicin and isoniazid, to ensure their precise administration and enhance the efficacy of treatment; (3) Cities exhibiting elevated levels of drug resistance warrant considerable attention, with a specific focus on promoting the rational and accurate utilization of antibiotics.

In recent years, nucleotide MALDI-TOF MS assay has been widely applied in various fields such as tumor, genetic diseases, bacterial and viral typing (11–13), including identification of MTB and its drug resistance (7, 14). Multiple studies have demonstrated that nucleotide MALDI-TOF MS assay performs well in TB diagnosis and drug resistance identification, with excellent sensitivity, specificity, and accuracy (15). Due to its high sensitivity, precision, high throughput, low cost, user-friendliness, and ease of analysis, more and more clinicians have recognized the clinical value of this technology. Moreover, China has released an expert consensus on the clinical application of nucleotide MALDI-TOF MS assay in TB and NTM diseases, recently (8). In this study, we utilized this technique to determine the etiology and drug resistance of clinically suspected TB patients in Shandong Province. The nucleotide MALDI-TOF MS assay was designed with multiple target genes for MTB identification, including commonly used genes such as *IS6110, IS1081*, and *gyrB* (16, 17), which to some extent increased the sensitivity of MTB detection. The results of this study showed a prevalence rate of MTB of 58.72% among suspected TB patients in Shandong Province, which is close to the reported MTB detection rate (59.3%) based on clinical diagnosis (18).

According to existing research reports, nucleotide MALDI-TOF MS assay exhibits high sensitivity, specificity, and concordance with phenotypic drug susceptibility testing results for various anti-TB drugs, especially for first-line drugs rifampicin and isoniazid (7). Our study shows that drug resistance to these two drugs among TB patients are both over 50%, possibly due to patients' non-compliant use of drugs or direct infection with drug-resistant MTB (19). In addition, a substantial number of patients have more complicated conditions and may also have a history of other infections for which similar antibiotics have been used. Currently, isoniazid mono-resistant TB is emerging as a growing global public health concern due to its association with poor treatment outcomes (20). The incidence of isoniazid mono-resistant TB is increasing progressively more frequently than rifampicin mono-resistant TB, and an estimated 11% of TB patients are isoniazid-resistant and rifampicin-sensitive, globally (21). Our study yielded similar results, showing that the incidence of isoniazid mono-resistant TB was 12.43%, slightly higher than the global estimate but also higher than that of rifampicin mono-resistant TB, suggesting that we should pay attention to the detection of isoniazid resistance. Additionally, it is noteworthy that the resistance rate to fluoroquinolones is also relatively high, ranking third after rifampicin and isoniazid, although the proportion of patients with monoreistance to it is not very high. Fluoroquinolones are widely used not only for treating TB but also for other types of infectious diseases (22), which might contribute to the high fluoroquinolone resistance rate. Considering that fluoroquinolones are core drugs for treating DR-TB patients, we need to pay attention to and strengthen monitoring of its drug resistance.

In this study, we also analyzed the geographical distribution of rifampicin-resistant cases, revealing that the three neighboring cities in the east had the highest number of cases, while Liaocheng in the west also contained 20 cases. We attempted to analyze *rpoB* mutations in the top six cities with the highest number of rifampicin-resistant cases. Among the four cities located in the east of Shandong Province, Weifang displayed the highest occurrence of mutated loci in *rpoB*, with mutations detected in all codons except for codon 513, which was exclusively found in Dezhou in the western region (Supplementary Figure 2B). Our results show that *rpoB* mutations that result in rifampicin resistance differed in different cities of Shandong Province.

Heteroresistance in TB has always been a matter of clinical concern (23), and there are differing opinions on the treatment of patients with heterogeneous DR-TB. Some believe that drugs corresponding to the resistant strains can still be used to kill the drug-resistant strains through increasing drug dosage. On the other hand, some argue that drugs corresponding to heterogeneous drug-resistant strains should not be continued, as doing so may exacerbate drug resistance, and under drug pressure, MTB could evolve into dominant drug-resistant strains. A consensus on how to treat patients with heterogeneous DR-TB has not been reached in clinical practice. Nucleotide MALDI-TOF MS assay has advantages in detecting heterogeneous drug resistance. Zhang et al. have reported this technology can detect 1% heteroresistance (24), reaching the level of phenotypic test, much higher than other common rapid molecular diagnostic methods, such as Xpert MTB/RIF, melting curve analysis and conventional sequencing (25). In addition, it is possible to achieve a detection limit of 0.1% through improvements and optimizations of nucleotide MALDI-TOF MS assay (26). Our results showed that MTB exhibited a certain proportion of heteroresistance to various anti-TB drugs, with rifampicin and fluoroquinolones the most significant rates of heteroresistance at 37.85 and 38.57%, respectively. Faye et al., have found the phenomenon of heteroresistance rate in the Rural Eastern Cape Province increased over time, from 9.6% in 2018 to 32.2% in 2020 (27). Recent study has characterized the trend of MTB drug resistance changes in a few TB patients during the treatment process and the proportion of drug-resistant strains indeed increases in some cases as treatment progresses, leading to complete drug resistance in later stages (22). If very low proportions of drug-resistant strains can be detected early and appropriate measures are taken to eliminate these strains, it may improve the success rate of subsequent treatment. Further related research is needed to confirm this.

Through nucleotide MALDI-TOF MS assay, we can also detect NTM infections. Our study showed that 14.86% of TB patients were infected with NTM, with *Mycobacterium intracellulare* being the leading species (data not shown), which is consistent with previous research findings (28). We also detected NTM infections in 52.06% of cases among non-TB group, indicating a relatively high NTM infection rate among suspected TB patients, which requires our attention. Indeed, with changes in people's lifestyles, and increase in sub-healthy and older-adult populations, and continuous progress in socio-economic and medical systems, the incidence of NTM infections has been on the rise, and NTM infected pulmonary diseases are gradually attracting clinical attention (29). Our results of this study suggest the risk of NTM infection among TB patients in Shandong Province, as well as the possibility of NTM infection among suspected TB patients, which should be given much attention, especially in cases with poor TB treatment outcomes.

The biggest strength of this study is its large sample size and multi-center prospective design, which provides a certain level of representativeness. However, we also acknowledge that the detection technology has some limitations. First, nucleotide MALDI-TOF MS assay is designed based on the current understanding of drug-resistant genes (30), and it is restricted to the detection of known drug-resistant loci. As a result, this study may have underestimated the resistance rates of certain drugs to some extent. Second, the assay does not systematically assess the accuracy of sample types other than respiratory specimens. However, the vast majority of samples included in this study were respiratory samples, making the impact more limited. Third, the assay was used for one sample per patient. However, the lab conducted three replicates to ensure accuracy for each sample.

In summary, this study utilized nucleotide MALDI-TOF MS assay to assess the MTB positivity rate and drug resistance among suspected TB patients in Shandong Province. The MTB positivity rate was found to be 58.72%, and the overall drug resistance rate was 62.68%, among which, the southwestern regions had lower MTB arbitrary drug resistance rates. The resistance rate for isoniazid and rifampicin were at high level, and fluoroquinolone drug resistance rate was also relatively high. Heteroresistance was common, with rifampicin and fluoroquinolones the most frequent. These results suggest that relevant authorities in Shandong Province should strengthen monitoring and management in cities with high MTB positivity rates and enhance drug resistance test for key drugs.

## Data availability statement

The raw data supporting the conclusions of this article will be made available by the authors, without undue reservation.

## Ethics statement

The studies involving humans were approved by the Ethics Committee of Public Health Clinical Center Affiliated to Shandong University. The studies were conducted in accordance with the local legislation and institutional requirements. Written informed consent for participation in this study was provided by the participants' legal guardians/next of kin.

## Author contributions

XG: Conceptualization, Methodology, Writing – original draft, Writing – review & editing. TL: Methodology, Writing – original draft. WH: Data curation, Writing – review & editing. YX: Data curation, Writing – review & editing. SX: Investigation, Methodology, Writing – review & editing. HM: Investigation, Methodology, Writing – review & editing. QW: Formal analysis, Writing – review & editing. QZ: Investigation, Methodology, Writing – review & editing. GY: Investigation, Methodology, Writing – review & editing. DX: Formal analysis, Writing – review & editing. PJ: Investigation, Methodology, Writing – review & editing. HW: Investigation, Methodology, Writing – review & editing. MLin: Investigation, Methodology, Writing – review & editing. MLiu: Investigation, Methodology, Writing – review & editing. MN: Investigation, Methodology, Writing – review & editing. DW: Investigation, Methodology, Writing – review & editing. YL: Investigation, Methodology, Writing – review & editing. LJ: Conceptualization, Data curation, Writing – review & editing. CD: Conceptualization, Methodology, Writing – original draft, Writing – review & editing. ZZ: Conceptualization, Data curation, Funding acquisition, Writing – review & editing.
