# Supplementary material for: The positivity rates and drug resistance patterns of Mycobacterium tuberculosis using nucleotide MALDI-TOF MS assay among suspected tuberculosis patients in Shandong, China: a multi-center prospective study
Source: Front Public Health. 2024 Jan 18;12:1322426. doi: 10.3389/fpubh.2024.1322426 (PMC10830759; doi:10.3389/fpubh.2024.1322426)
Supplement: Supplementary file 1 [file Table_1.docx]

Supplementary Material

# Supplementary Figures and Tables

## Supplementary Figures


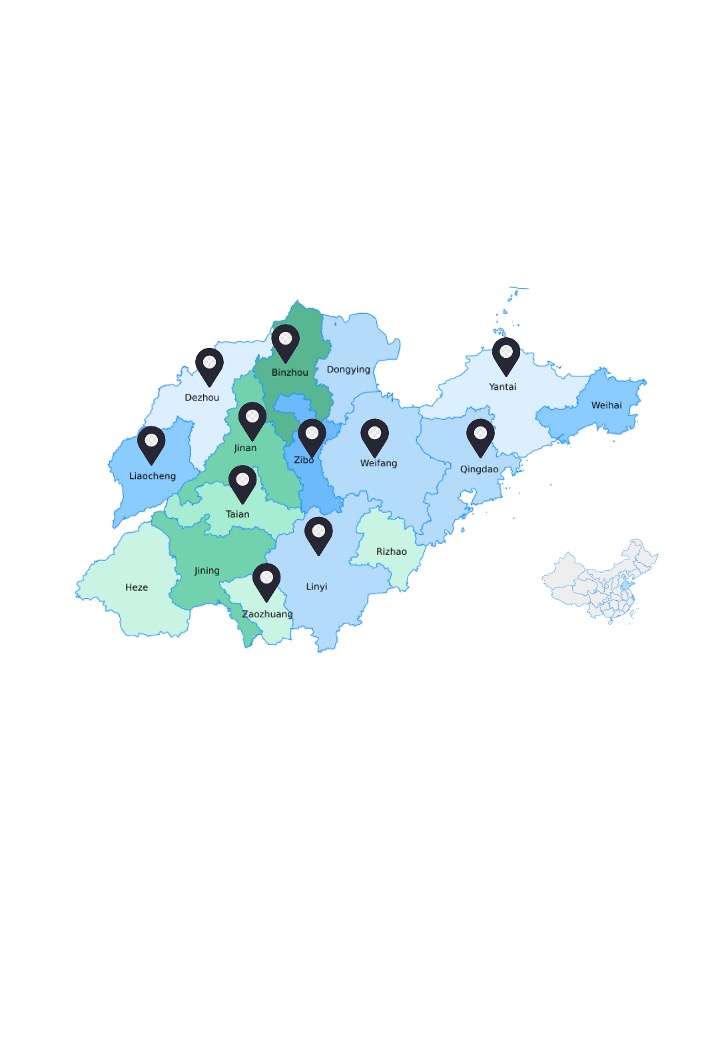


**Supplementary Figure 1.** Geographic distribution of located cities of participated hospitals in this study. A map of our country is presented in the lower right corner, with Shandong Province in the shaded area.


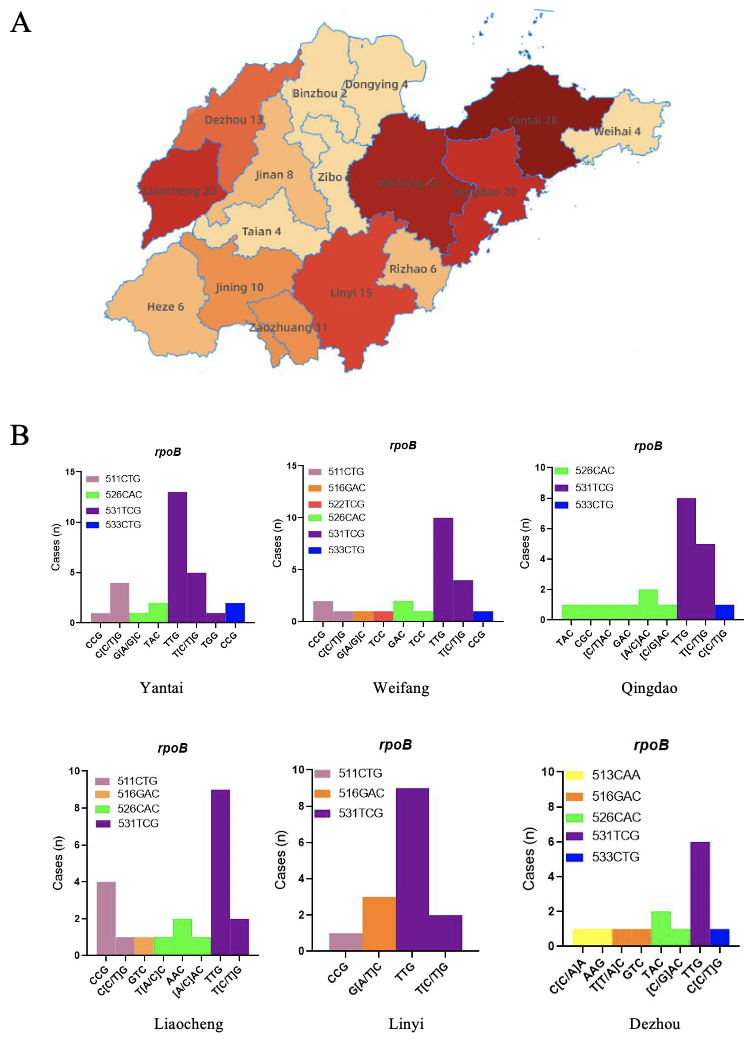


**Supplementary Figure 2.** The distribution of rifampicin-resistant cases and *rpoB* gene mutation loci in Shandong province. (A) Geographic distribution of rifampicin-resistant cases by city. (B) Distribution of resistance loci mutations for *rpoB* in the top six cities with the highest number of rifampicin-resistant cases, that is, Yantai, Weifang, Qingdao, Liaocheng, Linyi, and Dezhou cities.


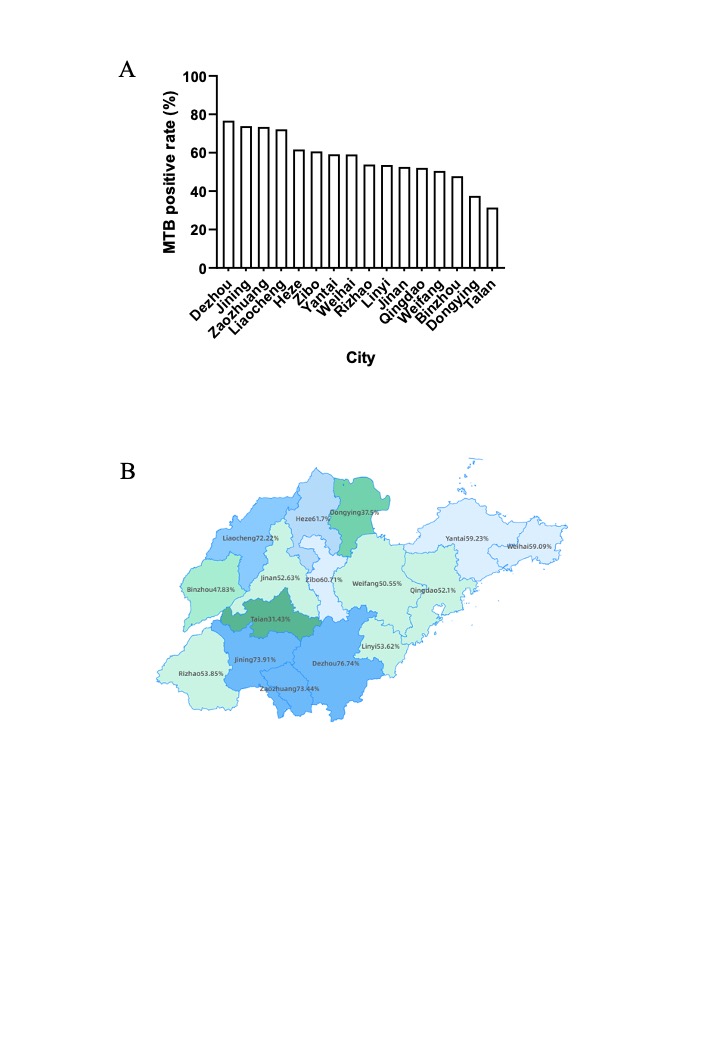


**Supplementary Figure 3.** The distribution of MTB positive rate in Shandong province. (A) Bars presenting the MTB positivity rate for each city of Shandong province. (B) Geographic distribution of MTB positivity rate by city.


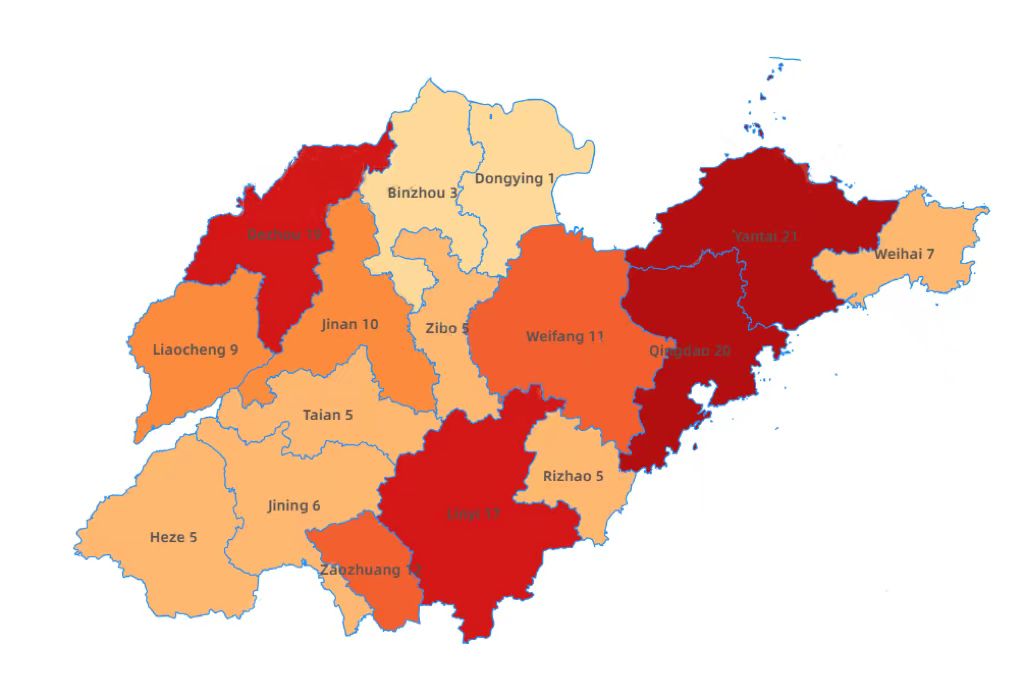


**Supplementary Figure 4.** Geographic distribution of TB patients resistant to three or more anti-TB drugs.

## Supplementary Tables

**Supplementary Table 1.** Information on drug-resistance gene loci detected by nucleotide MALDI-TOF MS.

| **Drug** | **Gene** | **Loci** | **Codon / Base** |
| --- | --- | --- | --- |
| Rifampicin  (RIF, R) | *rpoB* | 511 | CTG |
|  |  | 513 | CAA |
|  |  | 516 | GAC |
|  |  | 522 | TCG |
|  |  | 526 | CAC |
|  |  | 531 | TCG |
|  |  | 533 | CTG |
| Isoniazide  (INH, H) | *inhA* | -15 | C |
|  | *katG* | 315 | AGC |
|  |  | 316 | GGC |
| Pyrazinamide  (PZA, Z) | *pncA* | 57 | CAC |
| Ethambutol  （EMB, E） | *embB* | 306 | ATG |
|  |  | 406 | GGC |
| Fluoroquinolones  (FQs) | *gyrA* | 90 | GCG |
|  |  | 94 | GAC |
|  | *gyrB* | 538 | AAC |
| Streptomycin  （S） | *rpsL* | 43 | AAG |
|  |  | 88 | AAG |
| p-aminosalicylate (PAS) | *folC* | 43 | ATC |
|  | *thyA* | 202 | ACC |
|  |  | 75 | CAC |
| Amikacin  (Am) | *rrs* | 1401 | A |
|  |  | 1484 | G |
| Kanamycin (Km) | *eis* | -14 | C |
|  | *rrs* | 1401 | A |
|  |  | 1402 | C |
|  |  | 1484 | G |
| Capreomycin (Cm) | *rrs* | 1401 | A |
|  |  | 1402 | C |
|  |  | 1484 | G |
| Cycloserine (Cs) | *alr* | 261 | AGC |
| Clofazimine (CFZ) | *rv0678* | 193 | G |
|  |  | 466 | C |
| Bedaquiline (BDQ) | *rv0678* | 193 | G |
|  |  | 466 | C |
| Linezolid (LZD) | *rplC* | 460 | T |

**Supplementary Table 2.** Drug-resistance gene mutations of anti-TB drugs among DR-TB patients.

| **Gene** | **Loci** | **Case (n)** | **Wide-type Codon/bases** | **Wide-type amino acid** | **Mutant-type codon** | **Mutant-type amino acid** | **Case (n)** | **Percent (%)** |
| --- | --- | --- | --- | --- | --- | --- | --- | --- |
| *rpoB* | 511 | 18 | CTG | Leu | CCG | Pro | 11 | 3.18 |
|  |  |  |  |  | C[C/T]G | Leu/Pro | 7 | 2.02 |
|  | 513 | 3 | CAA | Gln | C[C/A]A | Pro/Gln | 2 | 0.58 |
|  |  |  |  |  | AAG | Lys | 1 | 0.29 |
|  | 516 | 13 | GAC | Asp | T[T/A]C | Phe/Tyr | 1 | 0.29 |
|  |  |  |  |  | G[A/T]C | Val/Asp | 6 | 1.73 |
|  |  |  |  |  | [G/T]AC | Tyr/Asp | 2 | 0.58 |
|  |  |  |  |  | G[A/G]C | Gly/Asp | 1 | 0.29 |
|  |  |  |  |  | GTC | Val | 3 | 0.87 |
|  | 522 | 0 | TCG | Ser | TCC | / | 0 | 0.00 |
|  | 526 | 24 | CAC | His | TAC | Tyr | 6 | 1.73 |
|  |  |  |  |  | CTC | Leu | 2 | 0.58 |
|  |  |  |  |  | T[A/C]C | Tyr/Ser | 1 | 0.29 |
|  |  |  |  |  | CGC | Arg | 2 | 0.58 |
|  |  |  |  |  | [C/T]AC | Tyr/His | 2 | 0.58 |
|  |  |  |  |  | C[A/G]C | Arg/His | 1 | 0.29 |
|  |  |  |  |  | AAC | Asn | 2 | 0.58 |
|  |  |  |  |  | GAC | Asp | 3 | 0.87 |
|  |  |  |  |  | TCC | Ser | 1 | 0.29 |
|  |  |  |  |  | [A/C]AC | Asn/His | 2 | 0.58 |
|  |  |  |  |  | [C/G]AC | Asp/His | 2 | 0.58 |
|  | 531 | 119 | TCG | Ser | TTG | Leu | 79 | 22.83 |
|  |  |  |  |  | T[C/T]G | Leu/Ser | 34 | 9.83 |
|  |  |  |  |  | TGG | Trp | 4 | 1.16 |
|  |  |  |  |  | CCG | Pro | 1 | 0.29 |
|  |  |  |  |  | T[C/G]G | Trp/Ser | 1 | 0.29 |
|  | 533 | 11 | CTG | Leu | C[C/T]G | Pro/Leu | 7 | 2.02 |
|  |  |  |  |  | CCG | Pro | 4 | 1.16 |
| *katG* | 315 | 147 | AGC | Ser | ACC | Thr | 93 | 26.88 |
|  |  |  |  |  | A[G/C]C | Thr/Ser | 44 | 12.72 |
|  |  |  |  |  | A[A/G]C | Asn/Ser | 6 | 1.73 |
|  |  |  |  |  | AG[C/G] | Arg/Ser | 1 | 0.29 |
|  |  |  |  |  | ATC | Ile | 2 | 0.58 |
|  |  |  |  |  | GGC | Gly | 1 | 0.29 |
|  | 316 | 2 | GGC | Gly | [A/G]GC | Ser/Gly | 2 | 0.58 |
| *inhA* | -15 | 44 | C | / | T | / | 39 | 11.27 |
|  |  |  |  |  | C/T | / | 5 | 1.45 |
| *pncA* | 57 | 5 | CAC | His | GAC | Asp | 4 | 1.16 |
|  |  |  |  |  | [C/G]AC | Asp/His | 1 | 0.29 |
| *embB* | 306 | 42 | ATG | Met | GTG | Val | 17 | 4.91 |
|  |  |  |  |  | ATA | Ile | 12 | 3.47 |
|  |  |  |  |  | ATC | Ile | 5 | 1.45 |
|  |  |  |  |  | ATT | Ile | 2 | 0.58 |
|  |  |  |  |  | CTG | Leu | 1 | 0.29 |
|  |  |  |  |  | [A/G]TG | Val/Met | 3 | 0.87 |
|  |  |  |  |  | [A/G]TA | Ile/Val | 1 | 0.29 |
|  |  |  |  |  | [A/G]T[A/G] | Val/Ile/Met | 1 | 0.29 |
|  | 406 | 9 | GGC | Gly | GAC | Asp | 7 | 2.02 |
|  |  |  |  |  | G[A/G]C | Asp/Gly | 2 | 0.58 |
| *gyrA* | 90 | 25 | GCG | Ala | G[C/T]G | Val/Ala | 11 | 3.18 |
|  |  |  |  |  | GAG | Glu | 1 | 0.29 |
|  |  |  |  |  | GTG | Val | 13 | 3.76 |
|  | 94 | 45 | GAC | Asp | GGC | Gly | 19 | 5.49 |
|  |  |  |  |  | GCC | Ala | 7 | 2.02 |
|  |  |  |  |  | G[A/G]C | Gly/Asp | 6 | 1.73 |
|  |  |  |  |  | G[A/T]C | Val/Asp | 9 | 2.60 |
|  |  |  |  |  | GTC | Val | 4 | 1.16 |
| *gyrB* | 538 | 3 | AAC | Asn | [A/G]AC | Asp/Asn | 3 | 0.87 |
| *rpsL* | 43 | 86 | AAG | Lys | AGG | Arg | 84 | 24.28 |
|  |  |  |  |  | A[A/G]G | Arg/Lys | 2 | 0.58 |
|  | 88 | 11 | AAG | Lys | AGG | Arg | 9 | 2.60 |
|  |  |  |  |  | A[A/G]G | Arg/Lys | 2 | 0.58 |
| *folC* | 43 | 0 | ATC | Ile | / | / | 0 | 0.00 |
| *thyA* | 202 | 1 | ACC | Thr | [A/G]CC | Ala/Thr | 1 | 0.29 |
|  | 75 | 11 | CAC | His | AAC | Asn | 8 | 2.31 |
|  |  |  |  |  | [A/C]AC | Asn/His | 3 | 0.87 |
| *rrs* | 1401 | 9 | A | / | A/G | / | 6 | 1.73 |
|  |  |  |  |  | G | / | 3 | 0.87 |
|  | 1402 | 1 | C | / | C/T | / | 1 | 0.29 |
|  | 1484 | 79 | G | / | G/T | / | 79 | 22.83 |
| *eis* | -14 | 1 | C | / | / | / | 1 | 0.29 |
| *alr* | 261 | 13 | AGC | Ser | AAC | Asn | 10 | 2.89 |
|  |  |  |  |  | A[A/G]C | Asn/Ser | 3 | 0.87 |
| *rv0678* | 193 | 2 | G | / | DEL | / | 2 | 0.58 |
|  | 466 | 3 | C | / | C/T | / | 2 | 0.58 |
|  |  |  |  |  | T | / | 1 | 0.29 |
| *rplC* | 460 | 2 | T | / | C | / | 2 | 0.58 |
